# Reduced Appendicular Lean Body Mass, Muscle Strength, and Size of Type II Muscle Fibers in Patients with Spondyloarthritis versus Healthy Controls: A Cross-Sectional Study

**DOI:** 10.1155/2016/6507692

**Published:** 2016-09-08

**Authors:** Kristine Røren Nordén, Hanne Dagfinrud, Amund Løvstad, Truls Raastad

**Affiliations:** ^1^Diakonhjemmet Hospital, National Unit for Rehabilitation in Rheumatology, Department of Rheumatology, Postboks 23 Vinderen, 0319 Oslo, Norway; ^2^Norwegian School of Sport Sciences, Sognsveien 220, 0863 Oslo, Norway; ^3^Diakonhjemmet Hospital, National Advisory Unit on Rehabilitation in Rheumatology, Postboks 23 Vinderen, 0319 Oslo, Norway

## Abstract

*Introduction*. The purpose of this study was to investigate body composition, muscle function, and muscle morphology in patients with spondyloarthritis (SpA).* Methods*. Ten male SpA patients (mean ± SD age 39 ± 4.1 years) were compared with ten healthy controls matched for sex, age, body mass index, and self-reported level of physical exercise. Body composition was measured by dual energy X-ray absorptiometry. Musculus quadriceps femoris (QF) strength was assessed by maximal isometric contractions prior to test of muscular endurance. Magnetic resonance imaging of QF was used to measure muscle size and calculate specific muscle strength. Percutaneous needle biopsy samples were taken from* m. vastus lateralis*.* Results*. SpA patients presented with significantly lower appendicular lean body mass (LBM) (*p* = 0.02), but there was no difference in bone mineral density, fat mass, or total LBM. Absolute QF strength was significantly lower in SpA patients (*p* = 0.03) with a parallel trend for specific strength (*p* = 0.08). Biopsy samples from the SpA patients revealed significantly smaller cross-sectional area (CSA) of type II muscle fibers (*p* = 0.04), but no difference in CSA type I fibers.* Conclusions*. Results indicate that the presence of SpA disease is associated with reduced appendicular LBM, muscle strength, and type II fiber CSA.

## 1. Introduction

The term spondyloarthritis (SpA) constitutes a group of related rheumatic diseases with ankylosing spondylitis (AS) often viewed as the prototype. Hallmark clinical features are inflammatory back pain, peripheral arthritis, and enthesopathy along with extra-articular manifestations such as uveitis, psoriasis and inflammatory bowel disease [1], and an increased risk of cardiovascular disease [2].

SpA is a systemic autoinflammatory disease, with abnormal production of proinflammatory cytokines by innate immune cells contributing to the pathogenesis [3]. Increased expression of TNF and other proinflammatory cytokines may curtail myogenic hypertrophic pathways and activate proteolytic pathways [4–6]. Patients with SpA often describe beneficial effects of physical activity (PA) on pain and stiffness, indicating a possible direct involvement of muscle tissue in SpA disease [7]. Inflammatory processes in skeletal muscle can rattle muscle homeostasis, although it is also recognized as a benign process in accelerating muscle repair and regeneration following injury or altered use [8, 9]. Notably, insight into muscle function and morphology in SpA is inconsistent and scarce. Lower muscle strength in SpA patients compared to healthy controls is reported [10–12], although normal level of muscle force is also described [13]. Regarding body composition, reduced lean body mass (LBM) and/or shifts in fat mass (FM) are observed in SpA patients compared to controls in a few cross-sectional studies [12, 14, 15], although discordant results have also been published [16–19]. At the level of local muscle tissue, dated studies observed mild to moderate histological abnormalities in AS patients [7, 20, 21]. Thus, there is a need for more knowledge regarding muscle morphology in SpA. Likewise, insight is also needed concerning the association between body composition, local muscle mass, and muscle strength in this patient population.

The purpose of this exploratory study was to test the hypothesis that the presence of SpA may be associated with reduced LBM and/or muscle strength. We further hypothesized that SpA patients display abnormal muscle morphology, including smaller muscle fibers and presence of inflammatory cells, in comparison to healthy controls.

## 2. Materials and Methods

### 2.1. Participants

A sample of 10 male patients with axial SpA according to the Assessment of SpondyloArthritis International Society (ASAS) classification criteria [22] were recruited from the outpatient clinic from the Rheumatology Unit at Diakonhjemmet Hospital, Oslo, and from patient organizations. Additional inclusion criteria were SpA symptom duration ≥5 years, age 30–45 years, and body mass index (BMI) (kg/m^2^) 18.5–30. Arthritis of the hip or knee joint or injury of the lower extremity in the past 12 months was considered exclusion criteria in addition to neuromuscular disease, cognitive impairment, and a current or previous history of peroral corticosteroid treatment. A convenient sample of 10 healthy controls fitting the same relevant inclusion criteria were enrolled in the study and pairwise matched to the patients by sex, BMI, and self-reported level of endurance and strength training. The study was approved by the Regional Ethics Committee and performed according to the Helsinki declaration. Participants were provided with oral and written information and signed a written consent form before enrolling in the study.

### 2.2. Subject Characteristics

All participants completed a questionnaire regarding age, level of education, occupational status, and smoking behavior. Habitual level of endurance and strength exercise was self-assessed on a four-graded ordinal scale. For the patients, Human Leukocyte Antigen (HLA) B27 phenotype, disease symptom duration, and time of diagnosis were registered and disease activity was evaluated by serum blood samples analyzed for C-reactive protein (CRP) and erythrocyte sedimentation rate (ESR). Bath Ankylosing Spondylitis Disease Activity Index (BASDAI), a self-reported questionnaire, was used as a measure of subjective disease activity [23]. Physical burden of disease was self-assessed by the Bath Ankylosing Spondylitis Functional Index (BASFI) [24]. Axial mobility was recorded using the 11-step Bath Ankylosing Spondylitis Metrology Index (BASMI) [25].

### 2.3. Measurements

#### 2.3.1. Body Composition

Body height and mass were recorded on a stadiometer (Seca 217, Seca gmbh & Co. kg., Hamburg, Germany) and used to calculate BMI. Dual energy X-ray absorptiometry (DXA) (Lunar iDXA, GE Healthcare, Buckinghamshire, United Kingdom) and enCORE Software (Version 14.10.022, GE Lunar) were used to estimate total and regional distribution of LBM and FM, including dispersion of android and gynoid fat. Appendicular skeletal muscle mass (RSMI) was predicted by the Baumgartner-equation [26]: RSMI = (tissue mass arms) + (tissue mass legs)/(height (m))^2^. To reduce the possibility of measurement error due to biological variation in hydration status [27, 28], participants were asked to fast for a minimum of four hours and refrain from exercise for 48 hours leading up to the DXA-scan.

#### 2.3.2. Quadriceps Femoris (QF) Volume and Cross-Sectional Area (CSA)

All participants underwent magnetic resonance imaging (Avanto TIM 76 × 18, Siemens, Erlangen, Germany) of the thighs. The scanning protocol consisted of three localizer scans for positioning, a T1-weighted turbo spin echo (TSE) sequence 15 slices of 5 mm thickness (1/15–15/15 of femur length), repetition time (TR) 432 ms, echo time (TE) 9.2 ms, field of view (FOV) 450 × 337.5 mm, voxel size 1 × 1 × 5 mm, and TSE factor 5. Fifteen images were produced by Siemens software (WB19, Siemens, Erlangen, Germany) and analyzed by a viewing tool (OsiriX Imaging Software, Pixmeo, Geneva, Switzerland).

Images numbers 2–13 were used in the data analyses. The perimeter of the muscle bellies in the anterior compartment of the thigh was manually outlined as region of interest (ROI). Interpolation at intervals of 1 : 15 femur length distance was used to estimate total volume of QF. Mean QF CSA was computed by averaging the CSA of all twelve ROIs and maximum QF CSA was determined by identifying the largest ROI captured.

#### 2.3.3. Muscle Function

Maximal voluntary contraction torque (MVC) of the knee extensors was measured at 90° knee angle using an isometric knee-extension device (GYM 2000 AS, Vikersund, Norway). Participants were stabilized in the apparatus with settings adjusted to ensure a 90° angle in the hip and knee joints. The lever arm of the knee-extension device with an attached force transducer (HBM U2AC2, Darmstadt, Germany) was adjusted so that the contact point with the participant's calf was just proximal to the line of the medial malleolus. Succeeding a warm-up protocol, the participants performed three unilateral maximal isometric contractions. Each contraction lasted for five sec, with 60 sec recovery time interspaced between each attempt. Participants were instructed to attain maximal force as quickly as possible and MVC torque (Newton meter—Nm) was recorded from the best trial (the highest MVC trial). LabView software (National Instruments Corporation, Austin, TX, USA) was used for data acquisition and inspection of time-force curves. The RFD data were smoothed with running average (20 ms) and the peak RFD (Nm/s) calculations were derived between 10 ms intervals (10th ms–1st ms). Specific muscle strength (maximal voluntary torque pr. unit muscle) was calculated as the MVC Nm/total QF volume ratio and MVC Nm/max QF CSA ratio. All MVC and RFD results are presented as the mean of the left and right leg, which were tested separately.

Following the MVC tests, the participants completed a test of muscular endurance. They performed as many repetitions of knee extension as possible with a resistance of 30% of MVC. Each repetition was performed at the pace of a metronome (Korg metronome MA-30, China) with one second in the concentric phase and one second in the eccentric phase. Initiating the movement with 90 degrees of flexion in the knee, the knee was fully extended and then lowered back to the starting position. Fatigue was defined as the inability to fully extend the knee at the pace of the metronome for three consecutive repetitions. A hand tally counter (Clas Ohlson, Insjön, Sweden) registered the total number of repetitions. Total workload (number of repetitions × kg) was then recorded. Unilateral measures of muscle function were computed and are presented as the total of right and left leg.

#### 2.3.4. Muscle Morphology

In an attempt to eliminate the acute effects of exercise on study variables [29], participants were asked to abstain from exercise for 48 hours prior to muscle tissue sampling. With participants in a supine position, the muscle biopsy was obtained under local anesthesia (Xylocaine + Adrenaline, 10 mg/mL + 5 *μ*g/mL; AstraZeneca, Södertälje, Sweden). A percutaneous biopsy was taken from the midsection of* m. vastus lateralis* using a 6 mm Pelomi needle (Albertslund, Denmark) with manual suction and a double-chop method. A total of approximately 100–150 mg tissue was collected. Following excision, tissue samples for immunohistochemical analyses were rinsed and embedded in Tissue-Tek compound (Cat # 4583, Sakura Finetek, CA, USA) prior to freezing in isopentane cooled on liquid nitrogen and thereafter stored at −80°C until further analysis.

*Immunohistochemical Analysis*. Tissue samples were thawed to −20°C and serial 8 *μ*m thick sections were cut using a microtome (CM 1860 UV, Leica Microsystems; Nussloch, Tyskland) and mounted on microscope slides (Superfrost Plus, Thermo Scientific, MA, USA). Muscle sections were double stained (see additional files in Supplementary Material available online at http://dx.doi.org/10.1155/2016/6507692) with antibodies against myosin heavy chain II (MHCII)/dystrophin, CD68/dystrophin, CD66b/dystrophin, and Pax7/laminin. Monoclonal antibodies against Pax7 were used to visualize satellite cells, CD68 and CD66b to visualize leukocytes (macrophages and neutrophils, resp.), SC-71 to visualize fiber type distribution (MHC II), and polyclonal antibodies against laminin and dystrophin to identify cellular position (Figures 5 and 6).

Slides were thereafter washed before incubation for 45 min with an appropriate secondary antibody (see additional files), washed again, coated with a coverslip and glued with ProLong Antifade Reagent with DAPI (Cat # P36935, Molecular Probes, Life Technologies, USA), and left to dry overnight at room temperature. A high-resolution camera (DP72, Olympus Corp., Tokyo, Japan) mounted on a microscope (BX61, Olympus Corp., Japan) with a fluorescent light source (X-cite 120PCQ; EXFO Photonic Solutions Inc., Mississauga, ON, Canada) was used to visualize and take pictures of the muscle sections. 

*Image Analysis*. The numbers of fibers and positive cells were manually counted. Muscle fibers cut longitudinally, fibers with fractured membranes, and fibers with unclear staining were excluded from further analysis. A total of 536 ± 183 and 577 ± 210 fibers were included in the analyses from the patients and the controls, respectively. The counted muscle fibers were used to determine the number of satellite cells, CD68 and CD66b positive cells per muscle fiber and per fiber phenotype. The analyses were performed on consecutive neighbor cross sections so that it was possible to identify fibers for the fiber type specific analyses of satellite cells.

The counting criterion for satellite cells was colocalization of Pax7 and DAPI staining within the laminin staining showed in Figure 5. Both intracellular and extracellular leukocytes (macrophages and neutrophils) were quantified by counting criterion of colocalization of DAPI staining and CD68 or CD66b and whether the positive cells were located inside or outside dystrophin staining (Figures 6(a) and 6(b)).

Myonuclei were analyzed with ImageJ using Fiji image and cell counter processing package (http://fiji.sc/Cell_Counter) (Figure 7(a)). To achieve satisfactory statistical power, 50 muscle fibers of each fiber type were included in the analysis [30]. DAPI staining with the geometric center within the dystrophin stain was defined as myonuclei (Figure 7(b)) [31]. Myonuclei are presented as number per muscle fiber and myonuclear domain was calculated by dividing the number of myonuclei by muscle fiber CSA.

TEMA (ChekVision, Hadsund, Denmark) was used to identify SC-71 staining for fiber type determination, and fiber CSA was analyzed by calculating the area within the dystrophin staining (Figure 7).

### 2.4. Statistical Analyses

Statistical analysis and tests were carried out using SPSS (version 21, IBM, Armonk, NY, USA) and GraphPad Prism (version 6, GraphPad Software, La Jolla, CA, USA). Descriptive statistics are presented as mean with standard deviation and minimum and maximum values. To analyze patient-control differences, normally distributed variables were compared using paired samples *t*-test. Skewed variables were log-transformed prior to parametric tests or analyzed by Wilcoxon signed ranks test. Ordinal variables were compared by Pearson chi square test. Significance was accepted at the 0.05-level. No systematic sample size calculation was performed by virtue of the pilot character of the study.

## 3. Results

### 3.1. Participant Characteristics and Disease Activity

Control participants were significantly taller and heavier than their patient fellow, although BMI was similar between groups. There was no difference between patients and controls in terms of age, employment status, smoking behavior, or level of endurance and strength exercise. Patients presented with a mean disease symptom and diagnosis duration of 15.5 ± 6.6 years and 10.0 ± 7.9 years, respectively, six patients had AS, one had psoriasis arthritis, and the remaining three presented with axial SpA (Table 1). One patient had previously undergone prosthetic replacement of the left hip due to congenital causes, and respective measures of muscle function and QF volume and CSA were excluded from all comparative analyses.

### 3.2. Body Composition

There was no significant group difference in bone mineral density (BMD), BMD *T*-score, total LBM, total body fat%, android fat%, or gynoid fat%. Appendicular lean mass (RSMI) was however significantly lower in the patient group (8.3 kg/m^2^) compared to the controls (8.8 kg/m^2^) (*p* = 0.02) (Table 1).

### 3.3. QF Volume and CSA

Total QF volume was significantly higher in control participants and may be accounted for by the coincidental inferior femur length/body height ratio in patients. Mean QF CSA was not significantly different between groups, but we observed a trend towards lower maximal QF CSA in the patients (*p* = 0.07) (Table 2).

### 3.4. Muscle Function

**Figure 1:**
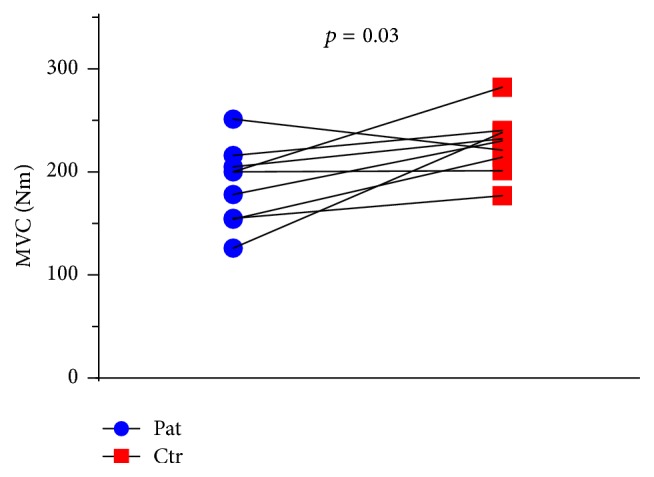
Maximal voluntary contraction (MVC) torque. Measured as isometric contraction of* m. quadriceps femoris*. Lines drawn signify the pairwise match of patient-control.

Between-group analyses of muscle function revealed no significant difference in RFD. The SpA patients had lower values for MVC (187 ± 38 Nm, Pat 01 omitted) compared to the controls (226 ± 29 Nm, Ctr 01 omitted) (*p* = 0.03, CI: −71.7; −5.9) (Figure 1). There was no significant difference in measures of specific strength when force was normalized to total QF volume. Muscle strength normalized to QF maximal CSA tended to be lower in the SpA patients (*p* = 0.08), although the difference was nonsignificant. Regarding muscle endurance, the number of repetitions at 30% MVC was practically identical (Table 2).

### 3.5. Muscle Morphology

#### 3.5.1. Fiber Type Distribution and Fiber CSA

**Figure 2:**
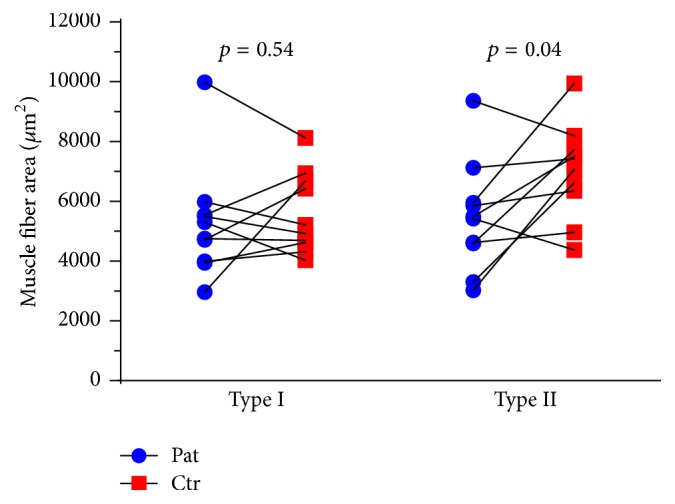
Muscle fiber CSA for type I and type II fibers in* m. vastus lateralis*. Lines drawn signify the pairwise match of patient-control.

Muscle fiber type distribution was similar between patients and controls, although the individual variation was large in both groups (Table 3). Mean type I fiber CSA was similar between patients (mean ± SD 5269 ± 1890 *μ*m^2^) and controls (5598 ± 1355 *μ*m^2^) (*p* = 0.54, CI: −1502.3; 884.3), whereas mean fiber CSA of type II fibers was 24.7% larger in controls (7019 ± 1585 *μ*m^2^) compared to patients (5474 ± 1840 *μ*m^2^) (*p* = 0.04, CI: −3281.0; −83.2) (Figure 2). Ratio of mean type II and type I fiber CSA was significantly higher in controls (Table 3).

#### 3.5.2. Myonuclei and Satellite Cells

**Figure 3:**
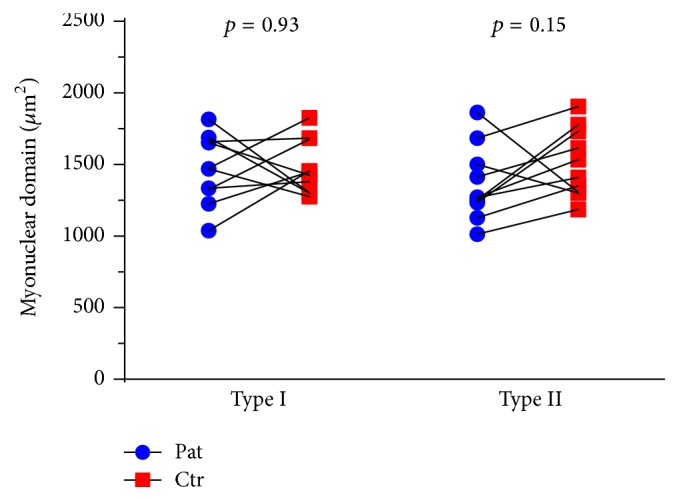
Myonuclear domain for muscle fiber type I and type II in* m. vastus lateralis*. Lines drawn signify the pairwise match of patient-control.

The mean number of myonuclei and central nuclei was comparable between groups. We detected a wide range in the number of satellite cells, with extreme values in both the patient and control group (Table 3). There was no group difference in satellite cells pr. type I fiber, whereas the tending difference in satellite cells for type II fibers was nonsignificant. Compared to controls, patients presented with a similar myonuclear domain for type I fibers (1469 ± 240 *μ*m^2^ versus 1478 ± 189 *μ*m^2^, *p* = 0.93, CI: −244.7; 226.1) and a nonsignificant tendency for lower type II fiber myonuclear domain (1351 ± 243 *μ*m^2^ versus 1512 ± 239 *μ*m^2^, *p* = 0.15, CI: −392.6; 70.2) (Figure 3).

#### 3.5.3. CD66b and CD68 Positive Cells

**Figure 4:**
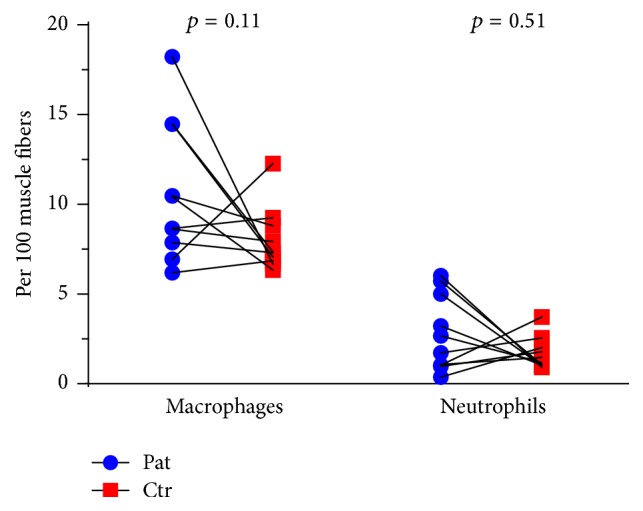
Number of CD68 positive macrophages and CD66b positive neutrophils in ECM in* m. vastus lateralis*. Lines drawn signify the pairwise match of patient-control; some lines appear duplicated as they represent pairs with similar values.

**Figure 5:**
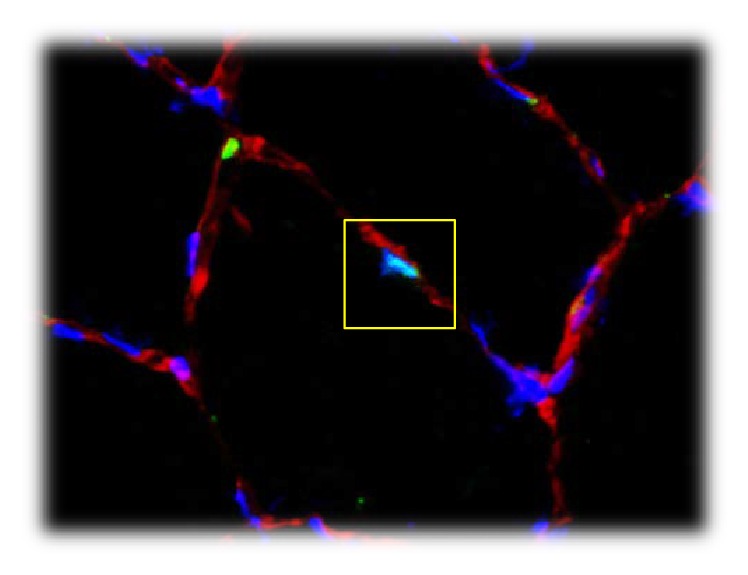
Pax7 positive cell (satellite cell) inside the yellow square. Red stain: laminin, green stain: Pax7, and blue stain: DAPI (nuclei).

**Figure 6:**
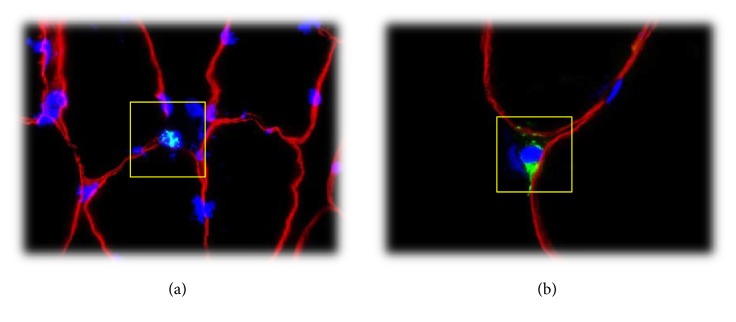
(a) CD66b positive cell (neutrophil granulocyte) inside the yellow square. Red stain: laminin, green stain: CD66b, and blue stain: DAPI (nuclei). (b) CD68 positive cell (macrophage) inside the yellow square. Red stain: laminin, green stain: CD68, and blue stain: DAPI (nuclei).

**Figure 7:**
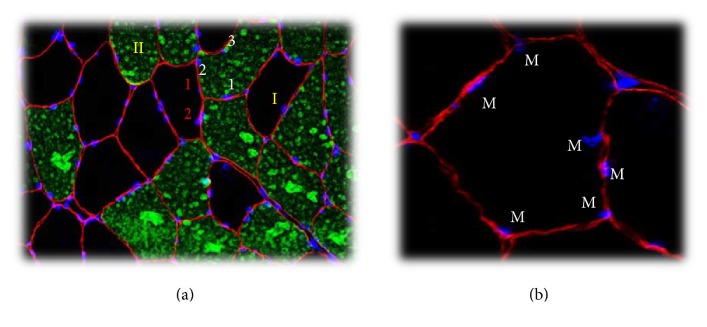
Counting myonuclei. (a) Biopsy marked with dystrophin (red stain), MHC II (green stain), and DAPI (blue stain). Roman numerals indicate fiber types I and II. Red numbers mark myonuclei in type I fibers and white numbers for type II fibers. (b) Blue stain: DAPI (myonuclei) and red markings: dystrophin. Myonuclei branded with M were determined to have their geometric center (nucleus center) inside the dystrophin labeling.

The amount of CD66b (marker for neutrophils) positive cells per 100 muscle fibers was similar (2.8 ± 2.1 and 1.7 ± 0.9 for patients and controls, resp.) and test of log-transformed variables was nonsignificant (*p* = 0.51, CI: −0.65; 1.21) (Figure 4). There was a tendency for increased level of CD68 (marker for macrophages) positive cells in patients (10.6 ± 3.9 per 100 fibers) compared to controls (7.9 ± 1.8), but the difference was nonsignificant (*p* = 0.11, nonparametric test) (Figure 4).

## 4. Discussion 

The main findings of the present study were a significantly lower appendicular LBM, muscle fiber type II CSA, and muscle strength in SpA patients compared to healthy controls. Furthermore, in the muscle biopsies of SpA patients, we detected a trend towards increased frequency of macrophages; heterogeneous leukocytes can both stimulate and attenuate muscle tissue repair [32].

Our observation of significantly lower appendicular LBM and no difference in total FM in SpA patients concur with the results of two previous studies [12, 15] but oppose no difference in body composition reported elsewhere [16–19]. Sari et al. [14] estimated body composition by methods of inferior validity [33] and detected lower FM in male AS patients compared to healthy controls. Comparison of body composition values between studies is challenging as most studies investigating SpA patients and healthy controls present absolute values of FM and LBM (kg) along with group differences in height and/or mass [12, 16, 18], thereby bypassing body composition values relative to stature (kg/m^2^).

We observed lower appendicular LBM in concert with significantly inferior muscle strength in the SpA patients. Direct comparison to other studies reporting analogous lower muscular force in AS patients [10–12] is challenging due to variation in measurement methods, contraction type, and muscle group studied. Furthermore, the reported group differences may have been confounded by disparity in training status between patients and control participants. In this study, patients were matched with a fellow control reporting corresponding levels of physical exercise. Nonetheless, we observed significantly lower knee extensor peak torque in the SpA patients. In contrast, a recent study [13] reported no difference in isometric knee extensor strength between AS patients and healthy controls with similar levels of PA, as well as comparable levels of muscle force values when normalized to ultrasound-derived measures of QF anatomical CSA (specific strength). Along with total muscle volume, QF maximal CSA is considered a strong predictor of muscle force [34]. Upon normalizing muscle force to QF maximal CSA (specific strength), we observed a tendency for lower specific strength in the patient group, indicating a possible difference in muscle quality.

An important observation in the current study is the significantly smaller type II fibers in the patient group. Myofiber size is positively correlated with muscle strength [35], and lower type II fiber CSA in SpA patients may explain the concomitant lower muscle strength. Fiber hypertrophy is further reported to enhance maximal torque and rate of torque development [35, 36] and measures of MVC and RFD are considered to be highly related [37]. The observation of no significant group difference in RFD despite lower maximum strength and smaller type II fibers in the SpA patients was therefore surprising. However, our RFD data shows large variance, and combined with a small sample size, measures of RFD may have been inflicted by a type 2 error.

Previous studies have detected angulated and atrophic muscle fibers in muscle biopsies yielded from AS patients [7, 20, 21]. However, myofiber CSA values and adequate matching to healthy controls are lacking in these studies, rendering it impossible to compare fiber size and study population to our current research. Disease manifestations may also differ from what we encounter in our present-day clinical practice due to modern therapeutic options. To our knowledge, ours is the first present-day study assessing muscle morphology in SpA. Strength training can induce preferential hypertrophy of type II fibers, whereas endurance exercise has limited effect on fiber size [38, 39]. We attempted to eliminate the influence of strength training on type II fiber size by matching patients and controls on self-reported exercise habits. Increased fiber CSA is associated with an initial enhancement in the ratio of myonuclei to cell cytoplasm volume [40], consistent with the observed trend towards greater type II fiber myonuclear domain in our control participants (Figure 3). On an opposite note, recent studies have highlighted the possibility that muscle atrophy is not accompanied by loss of myonuclei [41]. Although highly speculative, the trend towards decreased myonuclear domain in type II fibers from SpA patients may therefore illustrate the effect of catabolic pathways on previously enlarged muscle fibers.

Satellite cells are important for muscle regeneration and for hypertrophy in response to strength training [38]. While enhanced number of satellite cells has been observed following strength training [42, 43], proinflammatory pathways are associated with a decrease in volume and function of the satellite cell pool [8]. Interestingly, we observed a lower proportion of satellite cells in type II fibers in patients compared to controls. Although the group difference was nonsignificant (*p* = 0.13) (Table 3), we ponder on the possibility that inferior levels of satellite cells may curb the regenerative and/or adaptive ability of skeletal muscle in SpA patients. However, since the number of satellite cells may increase acutely several days after exercise [44], the 48-hour training ban may not have been sufficient to exclude exercise-driven changes in the number of satellite cells.

The present study detected a wide range in numbers of extracellular matrix (ECM) located macrophages, with a nonsignificant trend (*p* = 0.11) towards higher amounts in the biopsies from SpA patients. Inflammatory processes in muscle tissue are commonly observed following altered use or muscle injury but may also be a consequence of autoimmune skeletal muscle disease [4, 8]. The level of neutrophils was, however, comparable between patient and control participants. Biopsy samples were not stained for various macrophage phenotypes and we cannot estimate the proportion of pro- and anti-inflammatory macrophages. Proinflammatory macrophages secrete TNF and cytokines that may blunt myofiber regeneration, whereas anti-inflammatory macrophages promote differentiation of myogenic precursor cells [8, 45]. Study participants were advised to refrain from exercise the last two days prior to biopsy sampling. However, since the volume of macrophages is reported to plateau several days after exercise [46], the 48-hour training ban may not have been sufficient to exclude exercise-driven infiltration of macrophages. Nonetheless, the trend towards higher level of macrophages in the SpA patients may also be related to the disease in question. Ancillary immunoblot analysis of muscle biopsies is needed to identify whether the presence of SpA disease is associated with increased myogenic expression of factors known to curtail muscle hypertrophy or increase proteolysis.

Patients included in this study reported moderate current disease severity. Likewise, serum inflammatory values indicate low current disease activity compared to other studies with SpA patients [12, 14, 16, 17, 19, 47], although ESR and CRP presented with a wide range. However, patient self-report disclosed a history of disease flares in most patients, perhaps contributing to the observed difference in muscle function and morphology between patients and controls.

The effect of pharmaceutics commonly used in SpA on muscle tissue is equivocal and we cannot discard a confounding effect on study variables. Conducting a study on SpA patients with discontinued medication could perhaps unravel the true effect of disease on muscle tissue but involves inescapable ethical barriers. Conflicting results are reported in a systematic review [48] summarizing the effects of NSAIDs on parameters related to muscle growth. The majority of human trials do not uncover adverse effects of occasional use of NSAIDs on postexercise protein synthesis or muscle hypertrophy. Certain animal studies do however indicate the opposite [49, 50], and the consequence of longstanding use is unknown. Furthermore, evidence suggests that NSAIDs may blunt satellite cell activity and thus potentially curtail the potential of muscle hypertrophy [51]. Regarding TNF, prospective research has investigated the effect of initiating anti-TNF medication on parameters of body composition and the results are conflicting. Studies on SpA and psoriasis patients report shifts in body mass, FM, and/or LBM after commencing anti-TNF medication [52–55]. Discrepant results are reported in studies that noted no significant effect of TNF-blockers on body composition in patients with rheumatoid arthritis [56, 57]. There is a lack of human studies that histologically assess the impact of TNF-blockers on local muscle tissue. However, in mice with a genetic defect similar to dystrophy and myopathy, subcutaneous injections of TNF-blocker etanercept impeded inflammatory and degenerative histological changes [58], and low-dose infliximab was beneficial for muscle strength and muscle fibrosis [59]. Subgroup analysis of medication in our patient cohort is conducted on a small sample and should be interpreted with caution (data supplements). Collectively, our data may suggest a need for future SpA exercise studies to consider the potential effect of medication type on outcome variables.

There are several limitations to our study. Despite aiming to pairwise match patients and controls for level of endurance and strength exercise, an undisclosed group difference in training volume could have influenced study variables. Although one patient and his fellow control reported no leisure time exercise, our study may have been subject to selection bias by predominantly recruiting participants at the higher level of PA spectrum. In support, measures of maximal isometric muscle force in our study are comparable and slightly superior to values obtained using similar methods in a group of recreationally active young men and women [34]. Furthermore, mean myofiber CSA corresponds to normative morphology data for Norwegian males [60], although our study admitted individual patients and controls presenting with a mean fiber size comparable to power-lifters [61].

Researchers were not blinded, and although attempts were made to standardize all measurements, knowledge of group membership may have influenced the outcome variables. The limited number of participants may entail low statistical power, possibly inflating the chance of making type 2 errors. Because of the exploratory nature of the study, we have discussed group differences in muscle morphology that did not adhere to the* a priori* alpha-level of 0.05, and the observed trends need further investigation.

## 5. Conclusions

In conclusion, the present study indicates that the presence of SpA disease may be associated with reduced appendicular LBM, muscle strength, and type II fiber CSA. Further studies on larger and more diverse SpA cohorts are warranted to confirm our results.

## Supplementary Material

Supplementary Material includes primary and secondary antibodies used during immunohistochemical staining and supplementary statistical analyses (with-in group paired samples correlations and subject characteristics: NSAIDs vs TNF).

## Figures and Tables

**Table 1:** Subject characteristics^*∗*^.

Variable	Patients *n* = 10	Controls *n* = 10	*p* (95% CI lower; upper)^∧^
Age (at time of study)	39 ± 4.1 (33–44)	38 ± 5.1 (31–45)	0.57 (−3.7; 6.3)

Body height (cm)	178.1 ± 6.4 (169.5–190.5)	181.8 ± 6.3 (174.5–190.5)	0.04 (−7.3; −0.2)

Femur length	42.4 ± 2.4 (39.5–46.4)	45.4 ± 2.4 (42.5–49.5)	0.002 (−4.7; −1.4)

Femur length/body height (%)	24 ± 1 (23–25)	25 ± 1 (23–27)	0.01 (−0.02; −0.004)

Body mass (kg)	75.2 ± 7.8 (65.0–90.6)	79.9 ± 11.4 (74.2–95.5)	0.04 (−9.0; −0.3)

BMI, kg/m^2^	23.6 ± 1.3 (21.9–25.6)	24.0 ± 2.3 (20.0–27.8)	0.43 (−1.6; 0.7)

Smoking behavior			0.35^§^
Nonsmoker, *n* (%)	8 (80)	7 (70)	
Previous smoker, *n* (%)	1 (10)	3 (30)	
Current smoker, *n* (%)	1 (10)	0 (0)	

Full-time employment, *n* (%)	10 (100)	10 (100)	1.00

Level of endurance exercise (1–4)	2.3 ± 0.7 (1–3)	2.2 ± 0.6 (1–3)	0.89^§^

Level of strength exercise (1–4)	1.8 ± 0.6 (1–3)	2.0 ± 0.7 (1–3)	0.77^§^

HLA-B27 positive, *n* (%)	9 (90)		—

BASDAI score	2.7 ± 1.1 (0–3.7)		**—**

BASFI score	0.9 ± 0.8 (0–2.6)		**—**

BASMI score	2.3 ± 1.1 (0.7–4.6)		**—**

CRP, mg/L	7.0 ± 7.7 (<1–21)		**—**

ESR, mm/h	9.3 ± 9.7 (<1–26)		**—**

Anti-inflammatory medication			
NSAIDs, *n* (%)	6 (60)	0 (0)	—
DMARDs, *n* (%)	2 (20)	0 (0)	—
TNF-inhibitors, *n* (%)	4 (40)	0 (0)	—

BMD, g/cm^2^	1.27 ± 0.04 (1.20–1.33)	1.31 ± 0.13 (1.11–1.60)	0.39 (−0.15; 0.06)

BMD, *T-*score	0.65 ± 0.43 (0–1.3)	1.09 ± 1.33 (−0.9–4.0)	0.37 (−1.5; 0.6)

Lean body mass, kg/m^2^	18.2 ± 1.8 (15.1–21.3)	18.6 ± 1.6 (17.1–21.6)	0.32 (−1.2; 0.4)

Appendicular lean mass RSMI, kg/m^2^	8.3 ± 0.9 (7.2–9.6)	8.8 ± 0.8 (8.0–10.1)	0.02 (−0.9; −0.1)

Body fat, %	21.9 ± 7.0 (8.4–30.2)	20.1 ± 6.2 (12.6–31.2)	0.47 (−3.5; 7.1)

Android fat, %	25.3 ± 10.9 (6.3–39.7)	21.6 ± 10.1 (10.6–36.5)	0.31 (−4.1; 11.3)

Gynoid fat, %	21.0 ± 6.9 (8.2–29.6)	20.8 ± 5.7 (15.0–32.8)	0.95 (−5.3; 5.6)

^*∗*^Values are mean ± SD (range) unless otherwise indicated. ^∧^Comparison between patients and controls tested by paired samples *t*-test unless otherwise indicated. ^§^Pearson chi square test. AS: ankylosing spondylitis. BASDAI: Bath AS Disease Activity Index. BASFI: Bath AS Functional Index. BASMI: Bath AS Metrology Index. BMD: bone mineral density. BMI: body mass index. CI: confidence interval. CRP: C-reactive protein. DMARDs: disease modifying antirheumatic drugs. ESR: erythrocyte sedimentation rate. NSAIDs: nonsteroidal anti-inflammatory drugs. PsA: psoriasis arthritis. RSMI: relative skeletal muscle index. SpA: spondyloarthritis. TNF: tumor necrosis factor.

**Table 2:** Quadriceps femoris: parameters of volume, CSA, and muscle function^*∗*^.

Variable	Patients *n* = 9	Controls *n* = 9	*p* (95% CI lower; upper)^∧^
QF total volume, cm^3^	2013 ± 382 (1457–2738)	2296 ± 293 (1836–2705)	0.004 (−447.5; −118.4)

QF mean CSA, cm^2^	60 ± 9 (46–75)	63 ± 7 (52–72)	0.17 (−8.7; 1.8)

QF max CSA, cm^2^	81 ± 11 (65–97)	86 ± 9 (73–104)	0.07 (−12.3; 0.5)

Specific strength			
MVC Nm/QF total vol., Nm/cm^3^	0.094 ± 0.01 (0.08–0.11)	0.099 ± 0.01 (0.09–0.11)	0.36 (−0.017; 0.007)
MVC Nm/QF max CSA, Nm/cm^2^	2.32 ± 0.37 (1.87–2.93)	2.62 ± 0.22 (2.30–2.96)	0.08 (−0.64; 0.05)

Peak RFD, Nm/s	1639 ± 319 (1383–2425)	1950 ± 841 (1115–3913)	0.44^*£*§^

Muscle endurance			
Number of repetitions	38.3 ± 13.8 (19–56)	38.6 ± 8.2 (30–54)	0.95 (−13.6; 12.8)
Total workload (reps × kg), kg	568.5 ± 206.3 (267.8–857.2)	690.5 ± 151.4 (503.1–988)	0.10 (−270.4; 26.5)

^*∗*^Values are mean ± SD (range) unless otherwise indicated. ^∧^Comparison between groups tested by paired samples *t*-test unless otherwise indicated. ^*£*^Log-transformed variable. ^§^Wilcoxon signed ranks test. CSA: cross-sectional area. MVC: maximal voluntary contraction. Nm: Newton meter. QF: m. quadriceps femoris. RFD: rate of force development.

**Table 3:** Muscle morphology: parameters obtained from biopsy of *m. vastus lateralis* ^*∗*^.

Variable	Patients *n* = 10	Controls *n* = 10	*p* (95% CI lower; upper)^∧^
Muscle fiber distribution			
Type I, %	54.9 ± 13.8 (33.6–76.8)	48.5 ± 11.4 (30.5–68.3)	0.32 (−7.5; 20.5)
Type II, %	45.1 ± 13.8 (23.2–66.4)	51.5 ± 11.4 (31.7–69.5)	0.34 (−4.2; 10.8)

Ratio CSA type II/type I	1.05 ± 0.17 (0.83–1.37)	1.27 ± 0.22 (0.95–1.94)	0.05 (−0.442; −0.002)

Myonuclei			
Type I, pr. fiber	3.6 ± 1.1 (2.2–6.0)	3.8 ± 0.6 (3.1–4.8)	0.65 (−0.96; 0.63)
Type II, pr. fiber	4.0 ± 1.1 (2.6–6.0)	4.6 ± 1.0 (3.2–6.5)	0.25 (−1.75; 0.51)

Central nuclei			
Type I, pr. fiber	0.02 ± 0.03 (0.00–0.10)	0.08 ± 0.14 (0.00–0.44)	0.20^§^
Type II, pr. fiber	0.03 ± 0.03 (0.00–0.08)	0.08 ± 0.11 (0.00–0.32)	0.26^§^

Satellite cells, Pax7-positive			
Type I, pr. 100 fibers	8.8 ± 8.0 (1.5–29.7)	6.4 ± 4.2 (1.6–17.1)	0.62 (−0.61; 0.97)^*£*^
Type II, pr. 100 fibers	5.5 ± 2.7 (2.6–11.3)	8.0 ± 6.0 (3.4–24.3)	0.13 (−0.76; 0.12)^*£*^

^*∗*^Values are mean ± SD (range) unless otherwise indicated. ^§^Wilcoxon signed ranks test. ^*£*^Log-transformed variable, paired samples *t*-test. CD68: marker for macrophages. CD66b: marker for neutrophils. CSA: cross-sectional area. ECM: extracellular matrix. ^∧^Comparison between groups tested by paired samples *t*-test unless otherwise indicated.

## Abbreviations

AS: Ankylosing spondylitis
ASAS: Assessment of SpondyloArthritis International Society
BASDAI: Bath Ankylosing Spondylitis Disease Activity Index
BASFI: Bath Ankylosing Spondylitis Functional Index
BASMI: Bath Ankylosing Spondylitis Metrology Index
BMD: Bone mineral density
BMI: Body mass index
CRP: C-reactive protein
CSA: Cross-sectional area
DXA: Dual energy X-ray absorptiometry
ECM: Extracellular matrix
ESR: Erythrocyte sedimentation rate
FM: Fat mass
HLA: Human Leukocyte Antigen
LBM: Lean body mass
MHC: Myosin heavy chain
MVC: Maximal voluntary contraction
NSAIDs: Nonsteroidal anti-inflammatory drugs
OA: Osteoarthritis
QF: m. quadriceps femoris
RA: Rheumatoid arthritis
ROI: Region of interest
RFD: Rate of force development
RSMI: Appendicular lean body mass
SpA: Spondyloarthritis
TNF: Tumor necrosis factor.
