# Supplementary material for: Reduced Appendicular Lean Body Mass, Muscle Strength, and Size of Type II Muscle Fibers in Patients with Spondyloarthritis versus Healthy Controls: A Cross-Sectional Study
Source: ScientificWorldJournal. 2016 Sep 8;2016:6507692. doi: 10.1155/2016/6507692 (PMC5031855; doi:10.1155/2016/6507692)
Supplement: Supplementary file 1 — Supplementary Material includes primary and secondary antibodies used during immunohistochemical staining and supplementary statistical analyses (with-in group paired samples correlations and subject characteristics: NSAIDs vs TNF). [file 6507692.f1.docx]

**Additional file 1: Primary and secondary antibodies used during immunohistochemical staining.**

|  | Catalog number | Manufacturer | Host | Dilutions |
| --- | --- | --- | --- | --- |
| *Primary antibodies* |  |  |  |  |
| Pax7 | 1 ea 13/14/13 | DSHB | Mouse | 1:100 |
| SC71 | 1 11/8/12 | DSHB | Mouse | 1:100 |
| CD68 | M0718 | Dako | Mouse | 1:300 |
| CD66b | CLB – B13.9 | Saquin | Mouse | 1:500 |
| Laminin | Z0097 | Dako | Rabbit | 1:400 |
| Dystrophin | Ab 15277 | Abcam | Rabbit | 1:500 |
| *Secondary antibodies* |  |  |  |  |
| Alexa flour 488 | 20010 | Biotium | Goat antimouse | 1:200 |
| Alexa flour 594 | 20112 | Biotium | Goat antirabbit | 1:200 |

**Additional file 2: Supplementary statistical analyses**

**Table 1. Patient participants within-group paired sampled correlations of parameters of QF volume and CSA (*Pat 01 excluded from analysis)*.**

| Variable |  | QF total volume  left leg, cm^2^ | QF max CSA  left leg, cm^2^ | QF mean CSA left leg, cm^2^ |
| --- | --- | --- | --- | --- |
| QF total volume right leg, cm^2^ | Pearson’s *r*  *P* (2-tailed)  *N* | 0.97  <0.001  9 |  |  |
| QF max CSA right leg, cm^2^ | Pearson’s *r*  *P* (2-tailed)  *N* |  | 0.92  0.001  9 |  |
| QF mean CSA right leg, cm^2^ | Pearson’s *r*  *P* (2-tailed)  *N* |  |  | 0.96  <0.001  9 |

CSA: Cross-sectional area. QF: Mm. quadriceps femoris.

**Table 2. Control participants within-group paired sampled correlations of parameters of QF volume and CSA.**

| Variable |  | QF total volume  left leg, cm^2^ | QF max CSA  left leg, cm^2^ | QF mean CSA left leg, cm^2^ |
| --- | --- | --- | --- | --- |
| QF total volume right leg, cm^2^ | Pearson’s *r*  *P* (2-tailed)  *N* | 0.99  <0.001  10 |  |  |
| QF max CSA right leg, cm^2^ | Pearson’s *r*  *P* (2-tailed)  *N* |  | 0.98  <0.001  10 |  |
| QF mean CSA right leg, cm^2^ | Pearson’s *r*  *P* (2-tailed)  *N* |  |  | 0.99  <0.001  10 |

CSA: Cross-sectional area. QF: Mm. quadriceps femoris.

**Table 3. Patient participants within-group paired sampled correlations of parameters of QF function (*Pat 01 excluded from analysis)*.**

| Variable |  | RFD left leg, Nm/10 ms | MVC left leg, Nm | Number of repetitions, left leg | Total workload (reps x kg) left leg, kg | MVC Nm/QF total vol., left leg, Nm/cm^3^ | MVC Nm/QF max CSA, left leg, Nm/cm^2^ |
| --- | --- | --- | --- | --- | --- | --- | --- |
| RFD right leg, Nm/10ms | Pearson’s *r*  *P* (2-tailed)  *N* | 0.45  0.23  9 |  |  |  |  |  |
| MVC right leg, Nm | Pearson’s *r*  *P* (2-tailed)  *N* |  | 0.89  0.001  9 |  |  |  |  |
| Number of repetitions, right leg | Pearson’s *r*  *P* (2-tailed)  *N* |  |  | 0.94  <0.001  9 |  |  |  |
| Total workload (reps x kg) right leg, kg | Pearson’s *r*  *P* (2-tailed)  *N* |  |  |  | 0.85  0.04  9 |  |  |
| MVC Nm/QF total vol., right leg, Nm/cm^3^ | Pearson’s *r*  *P* (2-tailed)  *N* |  |  |  |  | 0.90  0.001  9 |  |
| MVC Nm/QF max CSA, right leg, Nm/cm^2^ | Pearson’s *r*  *P* (2-tailed)  *N* |  |  |  |  |  | 0.87  0.02  9 |

CSA: Cross-sectional area. MVC: Maximal voluntary contraction. Nm: Newton meter. QF: Mm. quadriceps femoris. RFD: Rate of force development.

**Table 4. Control participants within-group paired sampled correlations of parameters of QF function (*Pat 01 excluded from analysis)*.**

| Variable |  | RFD left leg, Nm/10 ms | MVC left leg, Nm | Number of repetitions, left leg | Total workload (reps x kg) left leg, kg | MVC Nm/QF total vol., left leg, Nm/cm^3^ | MVC Nm/QF max CSA, left leg, Nm/cm^2^ |
| --- | --- | --- | --- | --- | --- | --- | --- |
| RFD right leg, Nm/10ms | Kendall’s *τ*  *P* (2-tailed)  *N* | 0.33  0.18  10 |  |  |  |  |  |
| MVC right leg, Nm | Pearson’s *r*  *P* (2-tailed)  *N* |  | 0.95  <0.001  10 |  |  |  |  |
| Number of repetitions, right leg | Pearson’s *r*  *P* (2-tailed)  *N* |  |  | 0.78  0.007  10 |  |  |  |
| Total workload (reps x kg) right leg, kg | Pearson’s *r*  *P* (2-tailed)  *N* |  |  |  | 0.84  0.02  10 |  |  |
| MVC Nm/QF total vol., right leg, Nm/cm^3^ | Pearson’s *r*  *P* (2-tailed)  *N* |  |  |  |  | 0.83  0.003  10 |  |
| MVC Nm/QF max CSA, right leg, Nm/cm^2^ | Pearson’s *r*  *P* (2-tailed)  *N* |  |  |  |  |  | 0.78  0.07  10 |

CSA: Cross-sectional area. MVC: Maximal voluntary contraction. Nm: Newton meter. QF: Mm. quadriceps femoris. RFD: Rate of force development.

**Table 5. Subject characteristics: NSAIDs vs TNF***

| Variable | NSAIDs  *n*= 6 | TNF  *n*= 4 | *P* (95% CI lower; upper)^ |
| --- | --- | --- | --- |
| Age (as of 31.12.14) | 39 ±4.8 | 39 ±3.6 | 0.93 (-6.8;6.3) |
| BMI, kg/m^2^ | 23.4 ±1.3 | 24.0 ±1.5 | 0.50 (-2.6;1.4) |
| Diagnosis |  |  |  |
| AS, n | 4 | 2 | - |
| Axial SpA, n | 2 | 1 | - |
| PsA, n | 0 | 1 | - |
| HLA-B27 positive, n | 5 | 4 | - |
| Time since diagnosis, years | 9.5 ±10.5 | 10.8 ±2.2 | 0.82 (-13.8;11.3) |
| Duration of symptoms, years | 15.7 ±8.8 | 15.3 ±1.5 | 0.93 (-10.1;10.9) |
| BASDAI score | 3.1 ±0.4 | 2.2 ±1.6 | 0.15 (-0.51;0.09)£ |
| BASFI score | 1.3 ±0.8 | 0.4 ±0.5 | 0.11 (-0.2;1.9) |
| BASMI score | 2.2 ±1.4 | 2.6 ±0.7 | 0.61 (-2.2;1.4) |
| CRP | 11.7 ±6.4 | 0.0 ±0.0 | 0.007 (4.1;19.2) |
| ESR | 13.8 ±10.3 | 2.5 ±0.6 | 0.001 (1.0;2.6)£ |

Values are mean ±SD unless otherwise indicated. ^Comparison between patients on NSAIDs and TNF tested by independent samples t-test unless otherwise indicated. £: Log-transformed variable, independent samples t-test. AS: Ankylosing Spondylitis. BASDAI: Bath AS Disease Activity Index. BASFI: Bath AS Functional Index. BASMI: Bath AS Metrology Index BMI: Body mass index. CI: Confidence Interval. CRP: C-reactive protein. ESR: Erythrocyte sedimentation rate. NSAIDS: Non-steroidal anti-inflammatory drugs. TNF: Tumor Necrosis Factor. SpA: Spondyloarthritis. PsA: Psoriasis arthritis

**Table 6. Body composition analysis: NSAIDs vs TNF***

| Variable | NSAIDs  *n*=6 | TNF  *n=* 4 | *P* (95% CI lower; upper)^ |
| --- | --- | --- | --- |
| BMD, g/cm^2^ | 1.27 ±0.05 | 1.27 ±0.05 | 0.94 (-0.07;0.07) |
| BMD, *T-*score | 0.63 ±0.47 | 0.68 ±0.43 | 0.89 (-0.72;0.63) |
| Lean mass, kg/m^2^ | 17.9 ±2.4 | 18.5 0.45 | 0.58 (-3.1;1.9)& |
| Appendicular lean mass; RSMI, kg/m^2^ | 8.4 ±1.0 | 8.2 ±0.8 | 0.76 (-3.4;2.2) |
| Body fat, % | 20.8 ±9.1 | 23.6 ±1.9 | 0.49 (-12.3;6.7)& |
| Android fat, % | 23.7 ±13.6 | 27.6 ±5.8 | 0.60 (-20.8;12.9) |
| Gynoid fat, % | 20.2 ±8.7 | 22.2 ±3.4 | 0.67 (-12.8;8.7) |

*Values are mean ±SD unless otherwise indicated. ^Comparison between groups tested by independent samples t-test unless otherwise indicated. &: Equal variance not assumed. BMD: Bone mineral density. CI: Confidence Interval. RSMI: Relative skeletal muscle index.

**Table 7. QF – parameters of volume, CSA and muscle function: NSADIs vs TNF***

| Variable | NSAIDs  *n*=6(#*n*=5) | TNF  *n*=4 | *P* (95% CI lower; upper)^ |
| --- | --- | --- | --- |
| MVC right leg, Nm | 217 ±29 | 175 ±28 | 0.053 (-0.6;84.4) |
| MVC left leg, Nm# | 193 ±39 | 154 ±39 | 0.18 (-23;101) |
| QF total volume |  |  |  |
| Right leg, cm^2^ | 2163 ±441 | 1987 ±203 | 0.48 (-375;727) |
| Left leg, cm^2^# | 2039 ±495 | 1899 ±345 | 0.65 (-553;832) |
| QF mean CSA |  |  |  |
| Right leg, cm^2^ | 64 ±11 | 59 ±4 | 0.44 (-9;19) |
| Left leg, cm^2^# | 60 ±12 | 57 ±8 | 0.65 (-14;21) |
| QF max CSA |  |  |  |
| Right leg, cm^2^ | 85 ±13 | 82 ±8 | 0.71 (-14;19) |
| Left leg, cm^2^# | 80 ±14 | 77 ±12 | 0.71 (-17;24) |
| Specific strength |  |  |  |
| MVC Nm/QF total vol., right leg, Nm/cm^3^ | 0.10 ±0.02 | 0.09 ±0.01 | 0.14 (-0.01;0.03) |
| MVC Nm/QF total vol., left leg, Nm/cm^3^# | 0.10 ±0.01 | 0.08 ±0.01 | 0.08 (-0.0;0.03) |
| MVC Nm/QF max CSA, right leg, Nm/cm^2^ | 2.6 ±0.34 | 2.1 ±0.21 | 0.04 (0.01;0.90) |
| MVC Nm/QF max CSA, left leg, Nm/cm^2^# | 2.4 ±0.36 | 2.0 ±0.23 | 0.08 (-0.07;0.92) |
| RFD |  |  |  |
| Right leg, Nm/10 ms | 1869 ±451 | 1486 ±376 | 0.20 (-249;1015) |
| Left leg, Nm/10 ms# | 1611 ±408 | 1632 ±204 | 0.93 (-553;512) |
| Muscle endurance |  |  |  |
| Number of repetitions, right leg | 31 ±13 | 42 ±15 | 0.26 (-32;10) |
| Number of repetitions,  left leg# | 35 ±11 | 46 ±15 | 0.28 (-31;10) |
| Total workload  (reps x kg), right leg, kg | 537 ±224 | 586 ±206 | 0.73 (-373;275) |
| Total workload  (reps x kg), left leg, kg# | 548 ±169 | 583 ±294 | 0.83 (-401;332) |

*Values are mean ±SD unless otherwise indicated. #: *N=* 5 patients, Pat 01 excluded due to prosthesis left hip. ^: Comparison between groups tested by independent samples t-test unless otherwise indicated. £: Log-transformed variable, independent samples t-test. CSA: Cross-sectional area. MVC: Maximal voluntary contraction. Nm: Newton meter. QF: Mm. quadriceps femoris. RFD: Rate of force development.

**Table 8. Muscle morphology – parameters obtained from biopsy of *m. vastus lateralis:* NSAIDs vs TNF*****

| Variable | NSAIDs  *n*=6 | TNF  *n*=4 | *P*(95% CI lower; upper)^ |
| --- | --- | --- | --- |
| Muscle fiber distribution |  |  |  |
| Type I, % | 52 ±14 | 59 ±14 | 0.45 (-0.28;0.14) |
| Muscle fibre CSA |  |  |  |
| Type I, µm^2^ | 5483 ±2443 | 4948 ±730 | 0.87 (-0.47;0.54)£ |
| Type II, µm^2^ | 5692 ±2241 | 5149 ±1248 | 0.67 (-2328;3414) |
| Ratio CSA type II/ type I | 1.06 ±0.19 | 1.03 ±0.17 | 0.81 (-0.24;0.30) |
| Myonuclei |  |  |  |
| Type I, pr. fiber | 3.7 ±1.3 | 3.5 ±1.0 | 0.83 (-1.6;1.9) |
| Type II, pr. fiber | 4.3 ±1.2 | 3.7 ±1.0 | 0.46 (-1.1;2.3) |
| Myonuclear domain |  |  |  |
| Type I, µm^2^ | 1475 ±253 | 1460 ±258 | 0.93 (-364;394) |
| Type II, µm^2^ | 1308 ±216 | 1415 ±299 | 0.53 (-480;266) |
| Central nuclei |  |  |  |
| Type I, pr. fiber | 0.03 ±0.03 | 0.01 ±0.01 | 0.17§ |
| Type II, pr. fiber | 0.03 ±0.03 | 0.02 ±0.02 | 0.57 (-480;266) |
| Satellite cells, Pax7-positive |  |  |  |
| Type I, pr. 100 fibres | 6.6 ±3.9 | 11.9 ±11.9 | 0.38 (-1.71;0.72)£ |
| Type II, pr. 100 fibres | 6.3 ±3.0 | 4.2 ±1.9 | 0.24 (-1.7;6.0) |
| CD68 ECM, pr. 100 fibres | 10.5 ±3.3 | 10.9 ±5.2 | 0.89 (-6.5;5.7) |
| CD66b ECM, pr. 100 fibres | 3.3 ±2.5 | 2.0 ±1.3 | 0.37 (-1.9;4.5) |

*Values are mean ±SD unless otherwise indicated. §: Mann-Whitney U test. CD68: marker for macrophages. CD66b: marker for neutrophils. CSA: Cross-sectional area. ECM: Extracellular matrix. £: Log-transformed variable, independent samples t-test.
